# Safety, reactogenicity, and immunogenicity of a 12-valent pneumococcal non-typeable *Haemophilus influenzae* protein D-conjugate vaccine in healthy toddlers: results from a phase I, randomized trial

**DOI:** 10.1080/21645515.2020.1810493

**Published:** 2020-11-11

**Authors:** Michael Horn, Ulrich Behre, Magali Traskine, Kurt Dobbelaere, Dorota Borys

**Affiliations:** a Pediatric Office, Schönau Am Königssee, Germany; b Private Practice, Kehl, Germany; c HPV, Hepatitis and Pneumococcal Vaccines, Clinical R&D, GSK, Wavre, Belgium

**Keywords:** Pneumococcal conjugate vaccine, PHiD-CV, safety, booster dose, immunogenicity, toddlers

## Abstract

As a stepping stone toward evaluation in infants, the safety and immunogenicity of an investigational 12-valent pneumococcal non-typeable *Haemophilus influenzae* protein D-conjugate vaccine (12vPHiD-CV) was assessed in toddlers. 12vPHiD-CV contains CRM_197_-conjugated capsular polysaccharides of serotypes 6A and 19A in addition to capsular polysaccharides of the 10 serotypes in PHiD-CV. In this phase I, double-blind, multicenter study (NCT01485406) conducted in Germany, 61 healthy toddlers aged 12–23 months previously primed with three PHiD-CV doses were randomized (1:1) to receive one dose of 12vPHiD-CV or PHiD-CV. Safety and reactogenicity of 12vPHiD-CV were assessed in terms of occurrence of grade 3 vaccination-related solicited and unsolicited adverse events (AEs) and vaccination-related serious AEs. Immune responses were evaluated 1 month post-vaccination. Grade 3 solicited local AEs (all considered vaccination-related) were reported for two (6.5%, redness) and three (9.7%, swelling) toddlers in the 12vPHiD-CV group and one (3.4%, swelling) in the PHiD-CV group. Grade 3 vaccination-related solicited general AEs were only reported in the PHiD-CV group. No grade 3 unsolicited or serious AEs were reported. For PHiD-CV serotypes, 100% of toddlers in both groups had antibody concentrations ≥0.2 µg/mL 1 month post-vaccination, and antibody geometric mean concentrations increased from pre-boosting. For serotypes 6A and 19A, antibody responses tended to be higher in the 12vPHiD-CV than the PHiD-CV group. A single dose of 12vPHiD-CV administered in toddlers was well tolerated and no safety concerns were identified. Immune responses were comparable to those induced by PHiD-CV when administered in toddlers previously primed with three doses of PHiD-CV.

## Introduction

*Streptococcus pneumoniae* remains a leading cause of acute and/or invasive diseases, such as pneumonia, meningitis, invasive pneumococcal disease (IPD), and acute otitis media.^1–3^ This pathogen accounts for a significant proportion of deaths in children under 5 years of age, and more than 317300 deaths were estimated to have occurred globally, due to pneumococcal disease in 2015. Of note, most of these deaths occurred in four countries in Africa and Asia.^3^ However, worldwide mortality and morbidity have declined by approximately 51% since the introduction of pneumococcal conjugate vaccines (PCVs) in 2000.^3^

Immunization of young children against *S. pneumoniae* by inclusion of high-valent PCV in national immunization programs is recommended by the World Health Organization (WHO).^4^ Currently, two such PCVs are used globally: the 13-valent PCV (PCV13; *Prevnar 13/Prevenar 13*, Pfizer) and the pneumococcal non-typeable *Haemophilus influenzae* protein D-conjugate vaccine (PHiD-CV; *Synflorix*, GSK), which have been assessed to have a similar impact on overall IPD.^5–7^

In most countries, a high-to-very high PCV coverage (≥80%) has been achieved.^8^ Nonetheless, changes in serotype distribution occur over time, with emergence of non-vaccine serotypes (serotype replacement) being the main cause of IPD post-PCV implementation.^9–11^ To broaden coverage of IPD-causing serotypes and thereby tackle serotype replacement, polysaccharide conjugates for other pneumococcal serotypes could be added to the current formulations. However, this may influence the vaccine’s safety profile and/or increase the risk of immunological interference.^12^ Therefore, assessment in the vaccine’s target population is required.

An investigational vaccine 12vPHiD-CV, which contains CRM_197_-conjugated capsular polysaccharides of serotypes 6A and 19A in addition to components of PHiD-CV, has been developed and assessed. As for PHiD-CV, serotype 3 has not been added in the formulation due to observed lack of clinical efficacy against acute otitis media^13^ and the unclear clinical benefit offered. The current study evaluated the safety and immunogenicity of 12vPHiD-CV when administered as a single dose in toddlers aged 12–23 months previously primed with three doses of PHiD-CV as compared to a PHiD-CV booster dose. This study was conducted prior to assessment of 12vPHiD-CV in infants.^14^

## Methods

### Study design and participants

**Figure 1.**
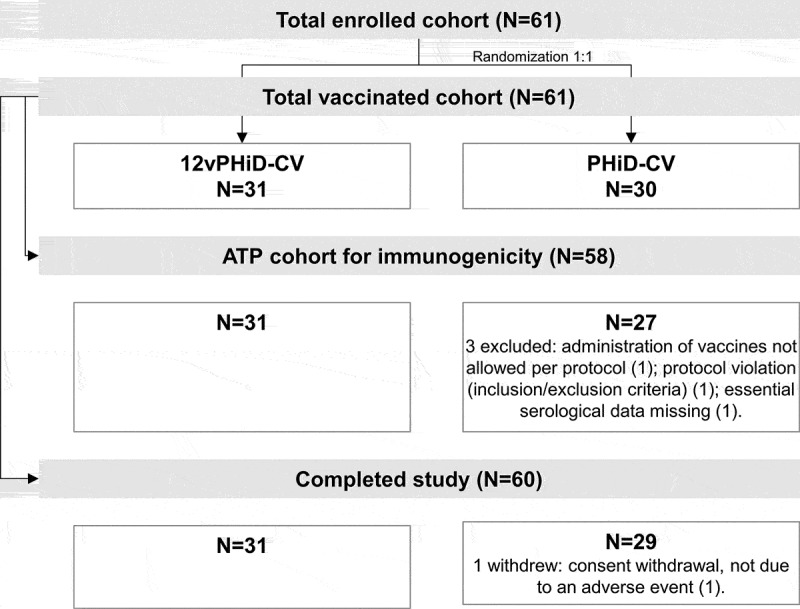
Participant flow chart

This phase I, randomized, controlled, double-blind study was conducted in four centers in Germany. Healthy toddlers 12–23 months of age were eligible for enrollment if they had received a full 3-dose vaccination course with PHiD-CV (outside this study) as reported by the investigator, were born after a gestation period of ≥36 weeks and if the investigator believed that their parent(s)/legally acceptable representative(s) (LARs) would comply with the requirements of the protocol. Children were randomized (1:1) to receive either 12vPHiD-CV or PHiD-CV (Figure 1). Randomization was performed using a web-based central randomization system, with a minimization algorithm accounting for the center.

N, number of toddlers; ATP, according-to-protocol.

The study was conducted in accordance with the Declaration of Helsinki and the principles of Good Clinical Practice. Informed consent was obtained prior to enrollment from children’s parents/LARs. The protocol, amendments, and informed consent forms were reviewed and approved by a national independent ethics committee. The study is registered at ClinicalTrials.gov (NCT01485406) and study details, including a full list of inclusion/exclusion criteria, are available at https://www.gsk-studyregister.com/(study ID 115373).

### Study objectives

The primary objective of the study was to evaluate the safety and reactogenicity of a single dose of 12vPHiD-CV administered to toddlers aged 12–23 months primed with PHiD-CV, in terms of occurrence of grade 3 vaccination-related solicited and unsolicited adverse events (AEs) and related serious AEs (SAEs). Secondary objectives included the assessment of safety in terms of any AE and SAE occurrence and evaluation of immune responses, 1 month post-vaccination.

### Vaccines

Vaccines (one 0.5 mL-dose) were administered intramuscularly in the deltoid of the non-dominant arm, or in the thigh if the deltoid muscle size was not adequate. Each dose of PHiD-CV contained 1 µg of each capsular polysaccharide for serotypes 1, 5, 6B, 7 F, 9 V, 14, and 23 F and 3 µg for serotype 4 conjugated to protein D (9–16 µg), 3 µg of capsular polysaccharide of serotype 18 C conjugated to tetanus toxoid (5–10 µg), 3 µg of capsular polysaccharide of serotype 19 F conjugated to diphtheria toxoid (3–6 µg), and 0.5 mg aluminum as aluminum phosphate. Each dose of 12vPHiD-CV contained in addition 2 µg of each capsular polysaccharide for serotypes 6A and 19A conjugated to CRM_197_ (4–10 µg).

### Safety and reactogenicity assessments

All events were reported by parents/LARs using diary cards. Solicited local and general AEs were recorded for 7 days post-vaccination (days 0–6). Unsolicited AEs and medically attended AEs were followed up for 31 days post-vaccination (days 0–30), and SAEs over the entire study period.

All events were graded by intensity on a 3-point scale, from 1 (mild) to 3 (severe). Grade 3 events were defined as “cried when limb was moved/spontaneously painful” for pain at injection site, “diameter >30 mm” for redness and swelling, “rectal temperature >40°C” for fever, “not eating at all” for loss of appetite, and “preventing normal activity” for all other AEs. Large swelling reactions (diameter >50 mm) following vaccination were also recorded.

All solicited local reactions were considered causally related to vaccination. The causality of all other AEs was assessed by the investigator.

### Immunogenicity assessments

Blood samples of approximately 5 mL were collected pre- and 1 month post-vaccination. Sera were stored at maximum −20°C prior to testing. Pneumococcal serotype-specific immunoglobulin G (IgG) antibodies were quantified by 22 F-inhibition enzyme-linked immunosorbent assay (ELISA),^15^ with a cutoff of 0.05 µg/mL. For each group, immune responses to each serotype were expressed as the percentage of toddlers with IgG concentrations ≥0.2 µg/mL, which are equivalent to concentrations of ≥0.35 µg/mL as measured by the non-22 F ELISA of the WHO reference laboratory,^16^ antibody geometric mean concentrations (GMCs) were also calculated. Opsonophagocytic activity (OPA) for antibodies against serotypes 6A and 19A was measured by a multiplex killing assay and expressed as the percentage of toddlers with titers equal or above the assay’s serotype-specific lower limit of quantitation (LLOQ; 151 for 6A and 143 for 19A) as well as geometric mean titers (GMTs). Of note, OPA was initially tested with a multiplex killing assay, but quality issues were subsequently detected, and remaining samples were re-tested with another multiplex OPA assay, leading to a lower number of available results. Anti-protein D antibodies were determined using an in-house ELISA, with a cutoff of 100 ELISA Units (EL.U)/mL, as previously described.^17^

### Statistical analyses

Given the vast experience with the vaccine components included in 12vPHiD-CV (i.e. CRM_197_-conjugates from PCV13 or conjugates from PHiD-CV), the study was designed with descriptive objectives, requiring a relatively small sample size (30 toddlers in each group). This sample size provided 80% power to detect an increase of 29.4–37.7% for the reporting rate of grade 3 and vaccine-related AEs in the 12vPHiD-CV group, assuming a true incidence of 2.0–20.0% in the PHiD-CV group. The sample size was calculated in PASS 2005, using the one-sided Chi-square test for two proportions with continuity correction and not adjusting for multiplicity (one-sided alpha = 0.025).

Safety analyses were carried out for the total vaccinated cohort. The percentages of toddlers reporting solicited and unsolicited AEs, AEs with causal relationship to vaccination, and grade 3 AEs were tabulated with 95% confidence interval (CI) for each group.

Immunogenicity analyses were performed for the according-to-protocol (ATP) cohort, including all evaluable toddlers who received the vaccine according to the protocol and who had data available at each timepoint. For each group, the percentages of toddlers with concentrations/titers above the pre-established thresholds were tabulated with two-sided 95% CIs. GMC/GMT calculations were performed by taking the anti-log of the mean of the log concentration/titer transformations. Antibody concentrations/titers below the assay cutoff/LLOQ were given an arbitrary value of half the cutoff/LLOQ. The 95% CIs for the GMTs/GMCs were obtained by exponential-transformation of the 95% CI for the mean of log-transformed titer/concentration (obtained by assuming that log-transformed values were normally distributed with unknown variance).

All statistical analyses were descriptive and were performed using SAS software, version 9.22 on SAS Drug Development.

## Results

### Demographics

The study was conducted between 12 December 2011 and 15 March 2012. Of the 61 toddlers vaccinated, 60 completed the study. In total, 58 toddlers (31 in the 12vPHiD-CV group and 27 in the PHiD-CV group) were included in the ATP cohort for immunogenicity (Figure 1).

The mean age at vaccination was 15.0 ± 2.75 months and most toddlers (98.4%) were of White/Caucasian-European heritage. There was a slight imbalance in sex distribution, with fewer boys enrolled in the PHiD-CV group (12/30; 40.0%) than in the 12vPHiD-CV group (15/31; 48.4%) (Table 1).

**Table 1.** Demographic characteristics for study participants (total vaccinated cohort)

	12vPHiD-CV group	PHiD-CV group	Total
	(N = 31)	(N = 30)	(N = 61)
Mean age at vaccination ± SD, months	15.5 ± 3.10	14.4 ± 2.27	15.0 ± 2.75
White/Caucasian-European heritage*, n (%)	30 (96.8)	30 (100)	60 (98.4)
White/Arabic-North African heritage*, n (%)	1 (3.2)	-	1 (1.6)
Male, n (%)	15 (48.4)	12 (40.0)	27 (44.3)

N, number of toddlers in each group; SD, standard deviation; n (%), number (percentage) of toddlers in each category.

Note: *Geographic ancestry data were collected from the children’s parents/legally acceptable representatives (sufficient information was provided by the study staff to answer this query correctly) and a questionnaire regarding ethnicity was filled in by the investigator.

### Safety and reactogenicity

**Figure 2.**
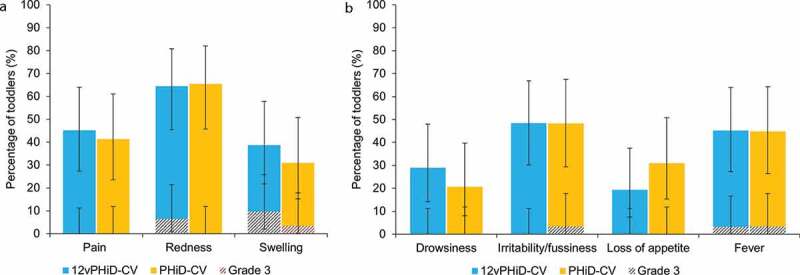
Percentage of toddlers with solicited local (A) and general (B) adverse events within the 7-day period following vaccination (total vaccinated cohort)

Note: Error bars represent 95% confidence intervals.

Following vaccination, the most frequently reported solicited local AE was redness, for 64.5% and 65.5% of toddlers in the 12vPHiD-CV and PHiD-CV groups, respectively. Grade 3 solicited local AEs were reported for two (6.5%) and three (9.7%) toddlers in the 12vPHiD-CV group (redness and swelling, respectively) and one (3.4%) child in the PHiD-CV group (swelling) (Figure 2, Supplementary Table 1).

Irritability/fussiness was the most frequently reported solicited general AE, for 48.4% of toddlers in the 12vPHiD-CV group and 48.3% of those in the PHiD-CV group. Grade 3 solicited general AEs were scarce, with severe fever reported in one (3.2%) child in the 12vPHiD-CV group and grade 3 irritability/fussiness and fever reported each in one (3.4%) child in the PHiD-CV group (Figure 2, Supplementary Table 1). Solicited general AEs related to vaccination were reported for 12.9–45.2% of toddlers in the 12vPHiD-CV group and 17.2–44.8% of toddlers in the PHiD-CV group. Grade 3 related solicited general AEs were reported only in the PHiD-CV group for irritability/fussiness and fever, each in one (3.4%) child (Supplementary Table 1). For each solicited AE, the reported local and general AEs were medically attended for at most one (3.2%) and five (16.1%) toddlers, respectively.

A large swelling reaction was reported for one child in the 12vPHiD-CV group, 1 day after vaccination. The reaction was limited to the injection site, not involving the adjacent joint and resolved without sequelae within 2 days.

At least one unsolicited AE was reported for 58.1% and 50.0% of toddlers in the12vPHiD-CV and PHiD-CV groups, respectively (Supplementary Table 1). The most frequent unsolicited AEs were rhinitis (in 12.9% of toddlers in the 12vPHiD-CV group and 6.7% of those in the PHiD-CV group) and viral infections (in 9.7% and 6.7% of toddlers in the12vPHiD-CV and PHiD-CV groups, respectively). Unsolicited AEs with causal relationship to vaccination were reported for one (3.2%) child in the 12vPHiD-CV group (malaise) and one (3.3%) in the PHiD-CV group (neurodermatitis). No grade 3 unsolicited AEs were reported. Medically attended unsolicited AEs were reported for 51.6% and 40.0% of toddlers in the 12vPHiD-CV and PHiD-CV groups, respectively.

No SAEs were reported during the study.

### Immunogenicity

Pre-vaccination, for each of the 10 PHiD-CV serotypes, ≥86.7% of toddlers in the 12vPHiD-CV group and ≥95.8% of toddlers in the PHiD-CV group had detectable antibody concentrations (≥0.05 μg/mL; data not shown), with ≥63.3% and ≥41.7%, respectively, having antibody concentrations ≥0.2 μg/mL (Table 2). For serotypes 6A and 19A, the percentage of toddlers with antibody concentrations ≥0.05 μg/mL were 86.7% (each) in the 12vPHiD-CV group, and 68.0% and 80.0%, respectively, in the PHiD-CV group; while for antibody concentrations ≥0.2 μg/mL, these percentages were 36.7% and 20.0% for serotype 6A and 66.7% and 36.0% for serotype 19A in the 12vPHiD-CV and PHiD-CV groups, respectively. One month post-vaccination, for each of the 10 PHiD-CV serotypes, all children in both groups had antibody concentrations ≥0.2 μg/mL. All toddlers in the 12vPHiD-CV group also had antibody concentrations ≥0.2 μg/mL for serotypes 6A and 19A, while in the PHiD-CV group, 96.2% and 96.3% had detectable antibody concentrations, with 84.6% and 96.3%, respectively, having concentrations ≥0.2 μg/mL.

**Table 2.** Percentages of toddlers with pneumococcal serotype-specific antibody concentrations ≥0.2 µg/mL and antibody GMCs, by timepoint (according-to-protocol cohort for immunogenicity)

		12vPHiD-CV				PHiD-CV		
		N	% (95% CI)	GMC (95% CI)		N	% (95% CI)	GMC (95% CI)
1	Pre	30	63.3 (43.9–80.1)	0.2 (0.1–0.3)		24	41.7 (22.1–63.4)	0.2 (0.1–0.3)
	Post	31	100 (88.8–100)	2.3 (1.7–3.1)		27	100 (87.2–100)	2.8 (2.1–3.8)
4	Pre	28	67.9 (47.6–84.1)	0.3 (0.2–0.5)		26	57.7 (36.9–76.6)	0.3 (0.2–0.4)
	Post	31	100 (88.8–100)	5.2 (4.1–6.6)		27	100 (87.2–100)	4.5 (3.3–6.1)
5	Pre	29	69.0 (49.2–84.7)	0.3 (0.2–0.5)		24	62.5 (40.6–81.2)	0.3 (0.2–0.5)
	Post	31	100 (88.8–100)	3.3 (2.5–4.4)		27	100 (87.2–100)	2.3 (1.5–3.5)
6B	Pre	30	66.7 (47.2–82.7)	0.3 (0.2–0.5)		24	41.7 (22.1–63.4)	0.2 (0.1–0.3)
	Post	31	100 (88.8–100)	5.4 (4.0–7.1)		27	100 (87.2–100)	1.9 (1.5–2.5)
7 F	Pre	30	86.7 (69.3–96.2)	0.8 (0.6–1.1)		24	95.8 (78.9–99.9)	0.7 (0.5–0.9)
	Post	31	100 (88.8–100)	3.8 (3.0–4.7)		27	100 (87.2–100)	3.4 (2.5–4.6)
9 V	Pre	30	90.0 (73.5–97.9)	0.7 (0.5–1.0)		24	83.3 (62.6–95.3)	0.6 (0.3–1.0)
	Post	31	100 (88.8–100)	4.4 (3.3–5.9)		27	100 (87.2–100)	3.6 (2.4–5.5)
14	Pre	30	93.3 (77.9–99.2)	0.9 (0.6–1.5)		24	91.7 (73.0–99.0)	0.8 (0.5–1.2)
	Post	31	100 (88.8–100)	8.3 (6.3–11.0)		27	100 (87.2–100)	7.2 (5.5–9.3)
18 C	Pre	30	83.3 (65.3–94.4)	0.7 (0.4–1.0)		24	79.2 (57.8–92.9)	0.6 (0.4–0.9)
	Post	31	100 (88.8–100)	11.3 (8.3–15.3)		27	100 (87.2–100)	11.2 (8.0–15.6)
19 F	Pre	30	83.3 (65.3–94.4)	1.2 (0.7–2.1)		24	91.7 (73.0–99.0)	0.6 (0.4–0.9)
	Post	31	100 (88.8–100)	17.3 (12.1–24.9)		27	100 (87.2–100)	10.9 (8.0–14.8)
23 F	Pre	30	70.0 (50.6–85.3)	0.4 (0.2–0.5)		24	75.0 (53.3–90.2)	0.4 (0.2–0.7)
	Post	31	100 (88.8–100)	3.5 (2.6–4.6)		27	100 (87.2–100)	3.5 (2.7–4.7)
6A	Pre	30	36.7 (19.9–56.1)	0.2 (0.1–0.3)		25	20.0 (6.8–40.7)	0.1 (0.1–0.1)
	Post	31	100 (88.8–100)	6.0 (3.9–9.1)		26	84.6 (65.1–95.6)	0.8 (0.4–1.3)
19A	Pre	30	66.7 (47.2–82.7)	0.3 (0.2–0.5)		25	36.0 (18.0–57.5)	0.2 (0.1–0.3)
	Post	31	100 (88.8–100)	8.2 (5.5–12.4)		27	96.3 (81.0–99.9)	2.0 (1.2–3.3)

N, number of toddlers with available results in each group; GMC, geometric mean concentration; CI, confidence interval; Pre, pre-vaccination; Post, 1 month post-vaccination.

For each pneumococcal serotype, antibody GMCs increased from pre- to 1 month post-vaccination in both groups. One month post-vaccination, antibody GMCs seemed to be similar between groups for all serotypes except for 6B (higher in the PHiD-CV group), 6A and 19A (higher in the 12vPHiD-CV group than in the PHiD-CV group) (Table 2).

Pre-vaccination, for serotypes 6A and 19A, the percentage of toddlers with OPA titers ≥LLOQ ranged between 25.0% and 50.0% in the two groups. One month post-vaccination, all toddlers in the12vPHiD-CV group and 82.4% and 95.0% in the PHiD-CV group, had OPA titers ≥LLOQ for serotypes 6A and 19A, respectively. For both serotypes, OPA GMTs increased from pre-vaccination levels to 1 month post-vaccination, with a more pronounced increase observed in the 12vPHiD-CV than the PHiD-CV group (Table 3).

**Table 3.** Percentages of toddlers with pneumococcal serotype-specific OPA titers above or equal to the assay serotype-specific LLOQ and OPA GMTs, by timepoint (according-to-protocol cohort for immunogenicity)

		12vPHiD-CV				PHiD-CV		
		N	% (95% CI)	GMT (95% CI)		N	% (95% CI)	GMT (95% CI)
6A	Pre	12	25.0 (5.5–57.2)	142.3 (66.4–304.8)		7	28.6 (3.7–71.0)	144.4 (51.8–402.1)
	Post	24	100 (85.8–100)	4515.3 (3426.5–5950.0)		17	82.4 (56.6–96.2)	1165.4 (571.8–2375.1)
19A	Pre	13	30.8 (9.1–61.4)	208.7 (73.0–596.7)		6	50.0 (11.8–88.2)	251.6 (55.2–1145.5)
	Post	25	100 (86.3–100)	5109.3 (3850.7–6779.4)		20	95.0 (75.1–99.9)	2796.3 (1708.7–4576.4)

OPA, opsonophagocytic activity; LLOQ, lower limit of quantitation; N, number of toddlers with available results in each group; GMT, geometric mean titer; CI, confidence interval; Pre, pre-vaccination; Post, 1 month post-vaccination.

Note: The assay LLOQ was 151 for serotype 6A and 143 for serotype 19A.

Observed immune response to protein D appeared similar between the two groups for both time points (Table 4).

**Table 4.** Percentages of toddlers with anti-protein D antibody concentrations ≥100 EL.U/mL and antibody GMCs, by timepoint (according-to-protocol cohort for immunogenicity)

	12vPHiD-CV				PHiD-CV		
	N	% (95% CI)	GMC (95% CI)		N	% (95% CI)	GMC (95% CI)
Pre	30	83.3 (65.3–94.4)	315.9 (209.5–476.4)		24	79.2 (57.8–92.9)	244.3 (153.7–388.3)
Post	31	96.8 (83.3–99.9)	1893.8 (1216.7–2947.6)		26	100 (86.8–100)	1493.0 (962.4–2316.1)

N, number of toddlers with available results; EL.U, enzyme-linked immunosorbent assay units; GMC, geometric mean concentration; CI, confidence interval; Pre, pre-vaccination; Post, 1 month post-vaccination.

## Discussion

A single dose of 12vPHiD-CV administered to healthy toddlers previously primed with three doses of PHiD-CV was well tolerated when compared to PHiD-CV and no safety concerns were raised.

In both groups, redness and pain were the most frequent local solicited AEs and irritability/fussiness was the most commonly reported general solicited AEs, similar to data previously reported for PHiD-CV following administration of a booster dose during the second year of life.^18,19^ Grade 3 local reactions, all considered related to vaccination, tended to have a higher incidence in the group receiving the investigational vaccine than in the group receiving PHiD-CV, but 95% CIs overlapped for the percentage of children experiencing each symptom. Grade 3 solicited general AEs related to vaccination were scarce and only occurred following vaccination with PHiD-CV. These comparisons should be interpreted cautiously in view of the small sample size for both groups.

The incidence of all solicited and unsolicited AEs was similar between toddlers receiving 12vPHiD-CV and PHiD-CV vaccination, and in line with a previous report in infants, following both a 3-dose 12vPHiD-CV primary course at ages 2, 3, and 4 months and a booster vaccination at 12–15 months of age.^14^ No SAEs occurred during the study and the one study withdrawal was not due to an AE. This study did not reveal any safety concern for 12vPHiD-CV vaccination in young children when compared to PHiD-CV vaccination, the latter being widely assessed in clinical trials and real-life settings.^19^

In line with previously reported immunogenicity results for a PHiD-CV booster dose administered following a two- or three-dose primary series in infants,^20–22^ one dose of either PHiD-CV or 12vPHiD-CV induced robust anamnestic immune responses against PHiD-CV serotypes in toddlers previously primed with three doses of PHiD-CV.

One month post-vaccination, immune responses against serotypes 6A and 19A tended to be higher in toddlers vaccinated with 12vPHiD-CV compared to those who received PHiD-CV, as expected due to the presence of the serotype-specific capsular polysaccharides in 12vPHiD-CV. Vaccination with PHiD-CV also elicits cross-reactive antibodies against the vaccine-related serotypes 6A and 19A. The immune responses against serotype 19A elicited by primary vaccination with PHiD-CV during infancy can be enhanced by the administration of a booster dose with a PCV containing this serotype. For instance, in a study comparing the immunogenicity of a PCV13 booster dose in toddlers previously receiving three PHiD-CV or PCV13 doses, antibody responses against 19A were non-inferior in PHiD-CV-primed compared to PCV13-primed toddlers.^23^ However, a PHiD-CV booster dose can also be used, considering the protective effectiveness of PHiD-CV against serotype 19A and a similar overall protection against IPD that was recently acknowledged for high-valent PCVs.^7^ Based on the evidence of the effectiveness of PHiD-CV against IPD caused by serotype 19A,^24–26^ the vaccine indication was updated to include cross-protection against this serotype.

As no safety concerns were identified in this study for 12vPHiD-CV in toddlers, and because the 6A and 19A CRM_197_-conjugates did not seem to have an impact on immunogenicity against PHiD-CV serotypes, the investigational vaccine was further evaluated in a subsequent study in infants. 12vPHiD-CV was shown to be immunogenic and well tolerated when administered as a 3 + 1 schedule, at 2, 3, 4, and 12–15 months of age, together with a routine pediatric combination vaccine.^14^

The study has several potential limitations. The sample size was relatively small, and as such only large differences in safety signals could be detected. The study was designed to provide a preliminary safety assessment of 12vPHiD-CV in healthy toddlers before vaccination in a younger age group, planned in a subsequent phase II trial. Because the study was focused on safety assessment, antibody functionality was only assessed for the additional serotypes 6A and 19A included in the investigational 12vPHiD-CV vaccine, in a limited number of toddlers from each group. Nevertheless, the study provided essential data prior to the assessment of 12vPHiD-CV in infants.^14^

In conclusion, a single dose of 12vPHiD-CV was well tolerated, with no safety concerns identified, and induced immune responses against the 12 vaccine pneumococcal serotypes and protein D when administered in toddlers 12–23 months of age, primed with three doses of PHiD-CV in infancy.

## Supplementary Material

Supplemental Material

## Trademark statement

*Synflorix* is a trademark licensed to the GSK group of companies. *Prevnar 13/Prevenar 13* is a trademark of Pfizer Inc. SAS Institute Inc. SAS and all other SAS Institute Inc. product or service names are registered trademarks or trademarks of SAS Institute Inc., Cary, NC, USA.
