# Supplementary material for: Safety, reactogenicity, and immunogenicity of a 12-valent pneumococcal non-typeable Haemophilus influenzae protein D-conjugate vaccine in healthy toddlers: results from a phase I, randomized trial
Source: Hum Vaccin Immunother. 2020 Nov 11;17(5):1463–9. doi: 10.1080/21645515.2020.1810493 (PMC8078718; doi:10.1080/21645515.2020.1810493)
Supplement: Supplemental Material [file KHVI_A_1810493_SM1429.docx]

**Supplementary material**

**Supplementary table 1.** Incidence of solicited adverse events reported within 7 days post-vaccination, unsolicited adverse events reported within 31 days post-vaccination and serious adverse events reported over the entire study period (total vaccinated cohort)

|  | % (95% CI) | | | |
| --- | --- | --- | --- | --- |
|  | 12vPHiD-CV (N=31) | |  | PHiD-CV (N=30) |
| *Solicited local AEs** |  |  |  |  |
| Pain | 45.2 (27.3–64.0) | |  | 41.4 (23.5–61.1) |
| Grade 3 | 0.0 (0.0–11.2) | |  | 0.0 (0.0–11.9) |
| Redness | 64.5 (45.4–80.8) | |  | 65.5 (45.7–82.1) |
| Grade 3 | 6.5 (0.8–21.4) | |  | 0.0 (0.0–11.9) |
| Swelling | 38.7 (21.8–57.8) | |  | 31.0 (15.3–50.8) |
| Grade 3 | 9.7 (2.0–25.8) | |  | 3.4 (0.1–17.8) |
| *Solicited general AEs* | | | | |
| Drowsiness | 29.0 (14.2–48.0) | |  | 20.7 (8.0–39.7) |
| Grade 3 | 0.0 (0.0–11.2) | |  | 0.0 (0.0–11.9) |
| Related | 25.8 (11.9–44.6) | |  | 17.2 (5.8–35.8) |
| Grade 3 related | 0.0 (0.0–11.2) | |  | 0.0 (0.0–11.9) |
| Irritability/fussiness | 48.4 (30.2–66.9) | |  | 48.3 (29.4–67.5) |
| Grade 3 | 0.0 (0.0–11.2) | |  | 3.4 (0.1–17.8) |
| Related | 45.2 (27.3–64.0) | |  | 44.8 (26.4–64.3) |
| Grade 3 related | 0.0 (0.0–11.2) | |  | 3.4 (0.1–17.8) |
| Loss of appetite | 19.4 (7.5–37.5) | |  | 31.0 (15.3–50.8) |
| Grade 3 | 0.0 (0.0–11.2) | |  | 0.0 (0.0–11.9) |
| Related | 12.9 (3.6–29.8) | |  | 17.2 (5.8–35.8) |
| Grade 3 related | 0.0 (0.0–11.2) | |  | 0.0 (0.0–11.9) |
| Fever | 45.2 (27.3–64.0) | |  | 44.8 (26.4–64.3) |
| Grade 3 | 3.2 (0.1–16.7) | |  | 3.4 (0.1–17.8) |
| Related | 32.3 (16.7–51.4) | |  | 37.9 (20.7–57.7) |
| Grade 3 related | 0.0 (0.0–11.2) | |  | 3.4 (0.1–17.8) |
| *Unsolicited AEs* |  |  |  |  |
| Any | 58.1 (39.1–75.5) | |  | 50.0 (31.3–68.7) |
| Grade 3 | 0.0 (0.0–11.2) | |  | 0.0 (0.0–11.6) |
| Related | 3.2 (0.1–16.7) | |  | 3.3 (0.1–17.2) |
| Grade 3 related | 0.0 (0.0–11.2) | |  | 0.0 (0.0–11.6) |
| Medically-attended | 51.6 (33.1–69.8) | |  | 40.0 (22.7–59.4) |
| Serious AEs | 0.0 (0.0–11.2) | |  | 0.0 (0.0–11.6) |

%, percentage of toddlers for whom at least one adverse event was reported; CI, confidence interval; N, number of children in each group; AE, adverse event.

Note: * All solicited local AEs were considered related to vaccination.
